# Analysis of potential categories and influencing factors of chronic disease comorbidity patterns among residents of Yantai City based on the health ecology model

**DOI:** 10.3389/fpubh.2025.1693025

**Published:** 2025-11-13

**Authors:** Yaqing Liu, Qi Cui, Sixian Du, Shan Zheng, Feng Jiang, Xu Yang, Liwen Gong, Chunming Ye

**Affiliations:** 1 School of Medicine and Health Management, Huazhong University of Science and Technology, Wuhan, Hubei, China; 2 Yantai Sports Industry Development Service Center, Yantai, Shandong, China

**Keywords:** chronic disease comorbidity, latent class analysis, health ecology model, Yantai City, health management

## Abstract

**Objective:**

To explore the latent category characteristics of chronic disease comorbidity among residents in Yantai City, Shandong Province, based on the theoretical framework of the health ecology model, and to identify key factors influencing different comorbidity patterns, thereby providing scientific basis for formulating targeted chronic disease prevention and control strategies.

**Methods:**

A cross-sectional survey was conducted in Yantai City in 2024 using convenience sampling to collect questionnaire data from 10,681 permanent residents aged 18 years and older. Latent class analysis identified typical comorbidity patterns among residents, followed by multinomial logistic regression to analyze factors influencing different comorbidity patterns.

**Results:**

Four chronic disease comorbidity patterns were identified among Yantai residents: (1) Low Comorbidity Group (C1, 82.39%), characterized by 1–2 chronic diseases; (2) Musculoskeletal-Chronic Disease Mixed Group (C2, 9.48%), characterized by coexisting musculoskeletal diseases and other chronic conditions; (3) Metabolic Syndrome-Dominant Group (C3, 7.01%), exhibiting clustering of metabolic disorders such as hypertension, diabetes, and dyslipidemia; (4) High Comorbidity-Complex Group (C4, 1.12%), involving complex combinations of three or more systemic chronic diseases. Multivariate logistic regression analysis revealed that increasing age was a common risk factor across all comorbidity groups (OR = 10.841, 95% CI: 8.853, 13.276). Gender effects exhibited pattern specificity: male gender was a protective factor for C2 (OR = 0.664, 95% CI: 0.552, 0.799) but a risk factor for C3 (OR = 1.745, 95% CI: 1.440, 2.116). At the behavioral level, regular physical exercise (OR = 0.755, 95% CI: 0.659, 0.865) and adequate sleep (OR = 0.437, 95% CI: 0.327, 0.583) were protective factors, while high-frequency consumption of pickled foods was a common risk factor (OR = 1.630, 95% CI: 1.387, 1.914), and alcohol consumption was a specific risk factor for Group C3 (OR = 1.425, 95% CI: 1.137, 1.785). Among socioeconomic factors, higher income levels (OR = 1.394, 95% CI: 1.096, 1.772) constituted a risk factor for Groups C2 and C3.

**Conclusion:**

Chronic disease comorbidity among Yantai residents exhibits significant population heterogeneity, categorizable into four distinct patterns. Influencing factors span multiple dimensions of the health ecology model. Public health practice should implement precision interventions, targeting high-risk populations with specific comorbidity patterns while integrating local dietary cultural characteristics. Tailored health promotion and disease management strategies are essential to effectively alleviate the burden of chronic disease comorbidity.

## 1 Introduction

Chronic noncommunicable diseases have become a major threat to global population health in the 21st century (1). With accelerating population aging, lifestyle changes, and improvements in healthcare standards, the prevalence of chronic diseases continues to rise worldwide, and their disease burden is increasingly severe (2). Against this backdrop, chronic disease comorbidity—as a complex health condition—has garnered extensive attention from both academia and public health practitioners (3). The World Health Organization explicitly defines chronic disease comorbidity as an individual’s simultaneous presence of two or more chronic conditions, encompassing common types such as cardiovascular disease, diabetes, chronic respiratory diseases, and malignant tumors (4). As a major and urgent challenge facing global public health today, chronic disease comorbidity not only directly impairs patients’ physical functions through pathophysiological interactions between diseases, leading to a significant decline in quality of life, but also markedly shortens patients’ life expectancy (5). Furthermore, individuals with chronic disease comorbidity typically require greater healthcare resources, including more frequent outpatient visits, longer hospital stays, and more complex long-term care needs, imposing substantial economic burdens and operational challenges on healthcare systems (6).

The health ecology model provides a multidimensional theoretical framework for analyzing the determinants of chronic disease comorbidity. Its core logic posits that individual health arises from the interplay of micro-level behaviors, social environments, and macro-level systems. By addressing multiple levels—individual, interpersonal, community, and policy—it offers a systematic analytical framework for understanding the complex etiology of chronic disease comorbidity (7). Specifically, this model typically encompasses the following levels: personal characteristics, behavioral traits, interpersonal networks, living and working conditions, and the policy environment. Extensive literature demonstrates that the health ecology model exhibits strong applicability and explanatory power in studies of chronic diseases and related comorbidities among older adults. It helps overcome the limitations of traditional single-factor analysis, revealing more comprehensively the intrinsic connections and pathways of influence among factors at various levels (8–10).

Conducting in-depth analyses of chronic disease comorbidity patterns in specific regions is a critical step toward achieving precision prevention and personalized health management (11). Differences in economic levels, geographical environments, lifestyles, and healthcare resource distribution across regions may shape unique chronic disease spectra and comorbidity patterns (12). However, current domestic and international research exhibits certain limitations, with many studies tending to utilize large-scale national public databases (e.g., CHARLS, CLHLS) for analysis (8, 11–13). While these studies offer advantages in large sample sizes and broad representativeness, they often struggle to reveal the subtle characteristics and unique risk factors of specific local populations. Consequently, research specifically examining population-level differences in chronic disease comorbidity patterns among Yantai residents remains relatively scarce.

Therefore, this study utilizes latent class analysis to examine the latent class structure of chronic disease comorbidity patterns based on Yantai City health survey data. Employing a health ecology model, it identifies the influencing factors of different comorbidity patterns through multivariate logistic regression. This approach aims to provide scientific evidence for developing precision health management strategies and optimizing comprehensive prevention and control measures for chronic diseases.

## 2 Materials and methods

### 2.1 Sources of information

This study recruited participants through convenience sampling among permanent residents of Yantai City, Shandong Province. Individuals meeting the following inclusion criteria were eligible to participate: age ≥18 years; continuous residence in Yantai City for 6 months or longer; full legal capacity and sound comprehension and communication abilities; and voluntary signing of the informed consent form after receiving full disclosure.

Exclusion criteria included: severe visual or hearing impairments preventing questionnaire completion or communication; mental illness or cognitive impairment hindering survey cooperation; and explicit refusal to participate. Survey announcements and questionnaire links were widely disseminated via online platforms such as official WeChat public accounts and resident health management groups to maximize coverage and accessibility. Data collection utilized the web-based “QuestionStar” platform questionnaire format. This study’s ethics approval number is: 2023S104.

The survey questionnaire comprised three core modules: (1) Socio-demographic characteristics: basic information including gender, age, residence, marital status, and living conditions; (2) Comprehensive health assessment: chronic disease prevalence, self-rated health status, and recent perceived psychological stress levels; (3) Physical exercise behavior and physical fitness evaluation: Recording the average number of days per week engaging in moderate-intensity or higher physical activity, the specific number of times per week using public sports and fitness facilities, and incorporating the overall evaluation results from the National Fitness and Health Monitoring Program.

During data processing, questionnaires with missing entries exceeding 10% of the total items were discarded entirely. For retained questionnaires meeting completeness requirements but containing sporadic missing values in continuous variables, multiple imputation methods were employed for filling.

### 2.2 Core variables

According to the health ecology model, the layers are arranged from the inside out as: personal characteristics layer, behavioral characteristics layer, interpersonal network layer, living and working conditions layer, and policy environment layer. However, since the “policy environment layer” estimates the impact on the entire population from a macro level, it cannot be defined at the individual level (14). Therefore, this study only incorporates factors potentially influencing the prevalence of chronic diseases among Yantai residents from four dimensions: personal characteristics, behavioral characteristics, interpersonal networks, and living and working conditions (15). (1) Personal characteristics layer: age, gender, place of residence. (2) Behavioral characteristics layer: weekly exercise frequency, weekly consumption frequency of preserved foods, smoking status, alcohol consumption status, daily sleep duration. (3) Interpersonal network layer: marital status, living alone status. (4) Living and working conditions layer: average monthly income, educational attainment, occupation. Specific values are shown in Table 1.

**Table 1:** Variables and their assignments.

Variable	Assignment
Gender	Female = 0, Male = 1
Age	18–39 years = 1, 40–59 years = 2, ≥60 years = 3
Residence	Rural = 0, Urban = 1
Exercise frequency	<2 days = 1, 2–3 days = 2, ≥4 days = 3
Pickled food consumption frequency	<2 days = 1, 2–3 days = 2, ≥4 days = 3
Smoking status	No = 0, Yes = 1
Alcohol status	No = 0, Yes = 1
Sleep duration	<6 h = 1, 6–8 h = 2, >8 h = 3
Marital status	Unmarried/divorced/widowed = 0, Married = 1
Living alone	No = 0, Yes = 1
Monthly income	<3,000 yuan = 1, 3,000–7,000 yuan = 2, >7,000 yuan = 3
Education level	Junior high school or below = 1, vocational/high school = 2, college diploma = 3, bachelor’s degree or above = 4
Occupation	Unemployed/other = 1, manual laborer = 2, non-manual worker = 3

### 2.3 Statistical analysis

#### 2.3.1 Descriptive analysis

This study employed Stata 18.0 and Mplus 8.3 statistical software for data analysis. First, descriptive statistical analysis was conducted on the basic characteristics of the included Yantai residents. Categorical variables were described using frequency (N) and composition ratio (%), aiming to clearly present the basic distribution of the study sample’s sociodemographic features. This provides a data foundation for subsequent latent category analysis and identification of influencing factors.

#### 2.3.2 Latent class analysis

This study employed Latent Class Analysis (LCA) to identify typical comorbidity patterns among the older adult(s) sample and statistically characterize chronic disease comorbidity patterns. LCA model fit indices included: (1) Akaike Information Criterion (AIC), Bayesian Information Criterion (BIC), and adjusted Bayesian Information Criterion (aBIC), which quantify model fit—lower values indicate better model fit; (2) Entropy: An indicator for model classification accuracy—values closer to 1 indicate higher classification accuracy, with values above 0.8 indicating classification accuracy exceeding 90%; (3) Likelihood ratio tests: including the Lo–Mendell–Rubin (LMR) test and bootstrap likelihood ratio test (BLRT). A *p*-value < 0.05 indicates that the k-category model outperforms the k-1-category model. When selecting the optimal model, consider not only the test metrics but also the model’s practical relevance and simplicity. Use χ^2^ tests for inter-category comparisons. Conduct multiple logistic regression with variables showing statistically significant differences in univariate analysis as independent variables to explore factors influencing different comorbidity patterns. Set the significance level α = 0.05.

## 3 Research findings

### 3.1 Demographic characteristics of surveyed patients

This study included a total of 10,681 subjects. In terms of gender, as shown in Table 2 females accounted for 68.78% (7,346 cases), significantly higher than males at 31.22% (3,335 cases). The age distribution was dominated by young and middle-aged individuals, with those aged 18–39 and 40–59 accounting for 44.53% (4,756 cases) and 43.85% (4,684 cases), respectively. In terms of urban–rural distribution, urban residents constituted 74.06% (7,910 cases), while rural residents comprised 25.94% (2,771 cases), indicating a higher concentration of urban samples. Regarding marital and living status, 81.64% (8,720 cases) were married, and 83.67% (8,937 cases) did not live alone, with “married and not living alone” being the predominant pattern; The highest proportion of economic income was in the 3,000–7,000 yuan/month range (53.96%, 5,764 cases). Educational attainment at the bachelor’s degree level or above accounted for 43.52% (4,648 cases). Among occupational distributions, non-manual workers constituted 52.73% (5,632 cases). These figures collectively reflect a tendency toward middle-income, higher-educated, and non-manual labor groups.

### 3.2 Identification of comorbidity patterns among Yantai residents

Using nine chronic disease indicators as manifest variables, five latent class models were established, as shown in Table 3. As the number of classes increased, AIC, BIC, and aBIC values gradually decreased, with the smallest decline observed at the five-class model. Considering classification accuracy, model interpretability, and sample size per latent class, the four-class model was selected as the optimal solution.

**Table 2:** Basic information of survey participants.

Variables	Number of examples		Percentage
Gender	Female	7,346	68.78%
	Male	3,335	31.22%
Age	18–39 years	4,756	44.53%
	40–59 years	4,684	43.85%
	≥60 years	1,241	11.62%
Residence	Rural	2,771	25.94%
	Urban	7,910	74.06%
Exercise frequency	<2 days	5,238	49.04%
	2–3 days	3,096	28.99%
	≥4 days	2,347	21.97%
Pickled food consumption frequency	<2 days	6,721	62.92%
	2–3 days	2,858	26.76%
	≥4 days	1,102	10.32%
Smoking status	No	9,573	89.63%
	Yes	1,108	10.37%
Alcohol status	No	9,090	85.10%
	Yes	1,591	14.90%
Sleep duration	<6 h	723	6.77%
	6–8 h	9,326	87.31%
	>8 h	632	5.92%
Marital status	Unmarried/divorced/widowed	1,961	18.36%
	Married	8,720	81.64%
Living alone	No	8,937	83.67%
	Yes	1,744	16.33%
Monthly income	<3,000 yuan	3,230	30.24%
	3,000–7,000yuan	5,764	53.96%
	>7,000 yuan	1,687	15.79%
Education level	Junior high school or below	1,526	14.29%
	Vocational/high school	1,961	18.36%
	College diploma	2,546	23.84%
	Bachelor’s degree or above	4,648	43.52%
Occupation	Unemployed/other	3,346	31.33%
	Manual laborer	1,703	15.94%
	Non-manual worker	5,632	52.73%

**Table 3:** Potential category model fitting indicators.

Model	LL	AIC	BIC	ABIC	Entropy	BLRT	VLMR	Category probability (%)
						*P* value	*P* value	
C1	−26652.514	53323.029	53388.515	53359.914	–			
C2	−23513.957	47065.915	47204.163	47143.783	0.826	<0.01	<0.01	0.15673/0.84327
C3	−23029.547	46117.095	46328.105	46235.947	0.837	<0.01	<0.01	0.01030/0.17807/0.81163
C4	−22749.998	45577.996	45861.769	45737.832	0.837	<0.01	<0.01	0.01114/0.07012/0.82389/0.09484
C5	−22644.100	45386.200	45742.735	45587.020	0.805	<0.01	<0.01	0.12115/0.05028/0.00749/0.02724/0.79384

**Figure 1:**
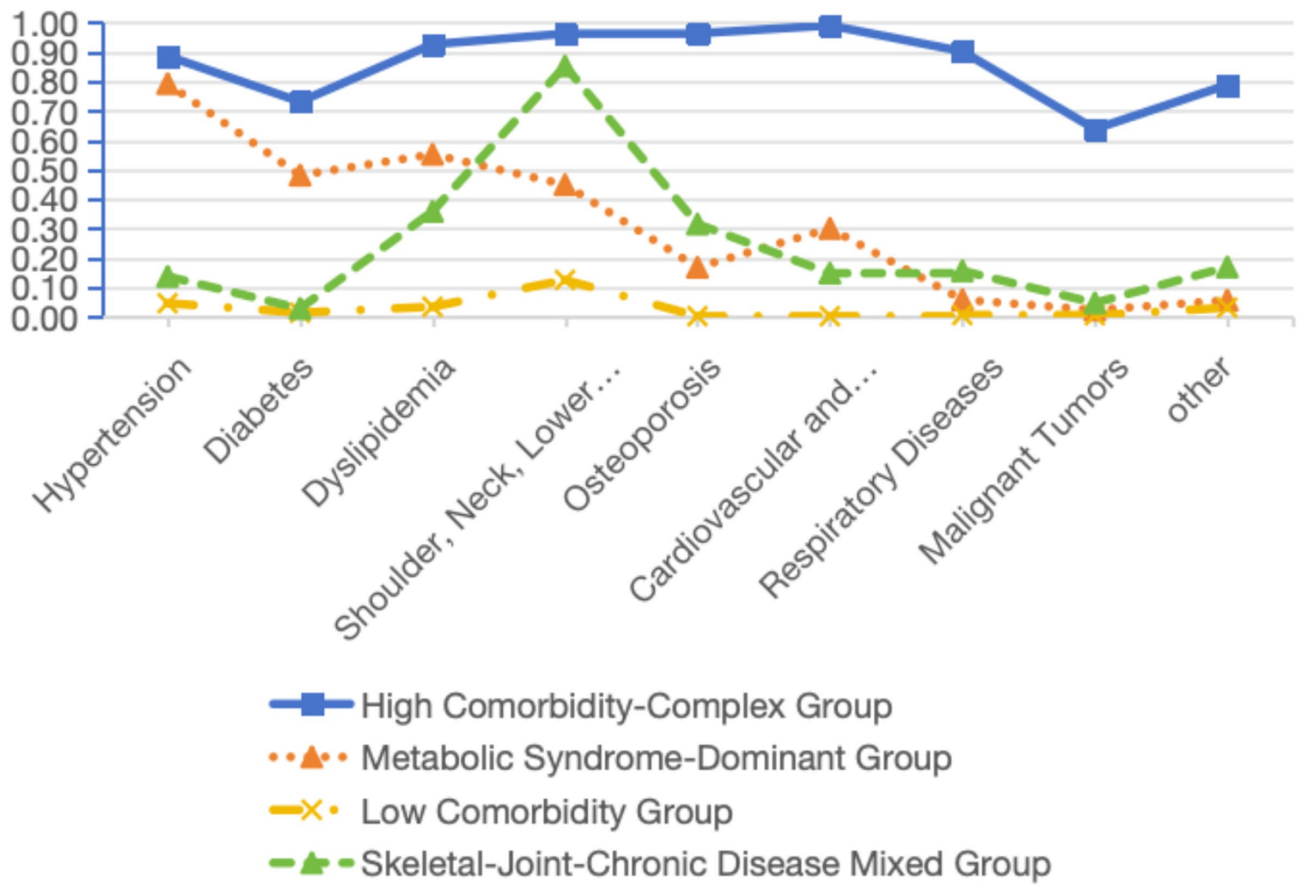
Distribution of the characteristics of the four potential categories of patients with chronic diseases.

The distribution of the four potential category characteristics is shown in Figure 1. Category 1 comprises 8,800 cases and exhibits the lowest overall probability scores across all four categories, hence named the “Low Comorbidity Group” (C1). Category 2 includes 1,013 cases and demonstrates higher levels in items 4 and 5, thus designated the “Musculoskeletal-Chronic Disease Mixed Group” (C2). Category 3 included 749 cases, exhibiting higher levels in items 1, 2, and 3, and was thus named the “Metabolic Syndrome-Dominant Group” (C3); Category 4 included 119 cases, exhibiting the highest levels across all items, and was thus named the “High Comorbidity-Complex Group” (C4).

### 3.3 Multivariate logistic analysis of factors influencing potential categories of comorbidity patterns among different patients

Using different categories of comorbidity combinations as the dependent variable, univariate analyses were conducted on the independent variables incorporated into the health ecology model. The results are presented in Table 4.

**Table 4:** Results of univariate analysis of different combinations of comorbidities in chronic disease.

Variables		C1 (*n* = 8,800)			C2 (*n* = 1,013)			C3 (*n* = 749)			C4 (*n* = 119)		
		Case (*n*%)	χ^2^	*P*	Case (*n*%)	χ^2^	*P*	Case (*n*%)	χ^2^	*P*	Case (*n*%)	χ^2^	*P*
Gender	Female	6,179 (70.22)	48.22	<0.01	748 (73.84)	13.36	<0.01	356 (47.53)	169.32	<0.01	63 (52.94)	14.05	<0.01
	Male	2,621 (29.78)			265 (26.16)			393 (52.47)			56 (47.06)		
Age	18–39 years	4,483 (50.94)	1,200	<0.01	194 (19.15)	293.92	<0.01	52 (6.94)	1,000	<0.01	27 (22.69)	40.68	<0.01
	40–59 years	3,626 (41.20)			634 (62.59)			365 (48.73)			59 (49.58)		
	≥60 years	691 (7.85)			185 (18.26)			332 (44.33)			33 (27.73)		
Residence	Rural	2,128 (24.18)	80.7	<0.01	305 (30.11)	10.11	<0.01	298 (39.79)	80.34	<0.01	40 (33.61)	3.69	0.05
	Urban	6,672 (75.82)			708 (69.89)			451 (60.21)			79 (66.39)		
Exercise frequency	<2 days	4,268 (48.50)	16.377	<0.01	523 (51.63)	4.642	0.098	376 (50.20)	10.886	<0.01	71 (59.66)	5.55	0.062
	2–3 days	2,623 (29.81)			265 (26.16)			182 (24.30)			26 (21.85)		
	≥4 days	1,909 (21.69)			225 (22.21)			191 (25.50)			22 (18.49)		
Pickled food consumption frequency	<2 days	5,670 (64.43)	93.598	<0.01	574 (56.66)	46.949	<0.01	410 (54.74)	32.18	<0.01	67 (56.30)	7.128	<0.05
	2–3 days	2,331 (26.49)			273 (26.95)			223 (29.77)			31 (26.05)		
	≥4 days	799 (9.08)			166 (16.39)			116 (15.49)			21 (17.65)		
Smoking status	No	7,967 (90.53)	44.28	<0.01	907 (89.54)	0.01	0.92	607 (81.04)	63.85	<0.01	92 (77.31)	19.63	<0.01
	Yes	833 (9.47)			106 (10.46)			142 (18.96)			27 (22.69)		
Alcohol status	No	7,584 (86.18)	45.76	<0.01	857 (84.60)	0.22	0.64	554 (73.97)	78.84	<0.01	95 (79.83)	2.64	0.1
	Yes	1,216 (13.82)			156 (15.40)			195 (26.03)			24 (20.17)		
Sleep duration	<6 h	464 (5.27)	178.21	<0.01	152 (15.00)	131.29	<0.01	90 (12.02)	35.833	<0.01	17 (14.29)	24.691	<0.01
	6–8 h	7,798 (88.61)			830 (81.93)			612 (81.71)			86 (72.27)		
	>8 h	538 (6.11)			31 (3.06)			47 (6.28)			16 (13.45)		
Marital status	Unmarried/divorced/widowed	1,741 (19.78)	67.64	<0.01	129 (12.73)	23.63	<0.01	69 (9.21)	44.97	<0.01	22 (18.49)	0.001	0.97
	Married	7,059 (80.22)			884 (87.27)			680 (90.79)			97 (81.51)		
Living alone	No	7,240 (82.27)	71.61	<0.01	911 (89.93)	32.09	<0.01	689 (91.99)	40.79	<0.01	97 (81.51)	0.41	0.52
	Yes	1,560 (17.73)			102 (10.07)			60 (8.01)			22 (18.49)		
Monthly income	<3,000 yuan	2,497 (28.38)	137.473	<0.01	352 (34.75)	50.064	<0.01	321 (42.86)	76.948	<0.01	60 (50.42)	23.358	<0.01
	3,000–7,000 yuan	4,978 (56.57)			445 (43.93)			294 (39.25)			47 (39.50)		
	>7,000 yuan	1,325 (15.06)			216 (21.32)			134 (17.89)			12 (10.08)		
Education level	Junior high school or below	1,014 (11.52)	417.93	<0.01	218 (21.52)	61.86	<0.01	267 (35.65)	386.46	<0.01	27 (22.69)	35.82	<0.01
	Vocational/high school	1,517 (17.24)			213 (21.03)			193 (25.77)			38 (31.93)		
	College diploma	2,186 (24.84)			200 (19.74)			128 (17.09)			32 (26.89)		
	Bachelor’s degree or above	4,083 (46.40)			382 (37.71)			161 (21.50)			22 (18.49)		
Occupation	Unemployed/other	2,771 (31.49)	228.163	<0.01	283 (27.94)	26.943	<0.01	245 (32.71)	237.149	<0.01	47 (39.50)	24.469	<0.01
	Manual laborer	1,192 (13.55)			218 (21.52)			259 (34.58)			34 (28.57)		
	Non-manual worker	4,837 (54.97)			512 (50.54)			245 (32.71)			38 (31.93)		

Taking different categories of comorbidity combinations as the dependent variable, the variables with statistical significance in the univariate analysis were included in the regression model. Logistic regression model was used for analysis, and the results are shown in Table 5.

**Table 5:** Logistic regression analysis results for different combinations of comorbidities.

Variables		C1			C2			C3			C4		
		*b*	OR	95%CI	*b*	OR	95%CI	*b*	OR	95%CI	*b*	OR	95%CI
Gender	Male	0.043	1.044	(0.911, 1.195)	−0.410***	0.664	(0.552, 0.799)	0.557***	1.745	(1.440, 2.116)	0.443	1.557	(0.999, 2.429)
Age	40–59 years	1.456***	4.290	(3.653, 5.037)	1.350***	3.857	(3.184, 4.674)	1.917***	6.801	(4.940, 9.361)	0.999***	2.716	(1.573, 4.687)
	≥60 years	2.383***	10.841	(8.853, 13.276)	1.809***	6.104	(4.691, 7.940)	3.281***	26.602	(18.774, 37.673)	1.807***	6.092	(3.205, 11.586)
Residence	Urban	−0.040	0.961	(0.844, 1.094)	−0.009	0.991	(0.840, 1.170)	−0.125	0.882	(0.730, 1.067)	0.199	1.220	(0.791, 1.883)
Exercise frequency	2–3 days	−0.205***	0.815	(0.715, 0.929)	−0.168**	0.845	(0.719, 0.995)	−0.200**	0.819	(0.670, 0.999)	−0.491**	0.612	(0.386, 0.971)
	≥4 days	−0.281***	0.755	(0.659, 0.865)	−0.257***	0.773	(0.650, 0.919)	−0.224**	0.799	(0.655, 0.975)	−0.712***	0.491	(0.300, 0.801)
Pickled food consumption frequency	2–3 days	0.163**	1.177	(1.039, 1.333)	0.155	1.168	(0.997, 1.366)	0.179*	1.196	(0.994, 1.439)	0.107	1.113	(0.720, 1.720)
	≥4 days	0.489***	1.630	(1.387, 1.914)	0.564***	1.758	(1.444, 2.139)	0.341***	1.406	(1.107, 1.788)	0.594**	1.811	(1.090, 3.006)
Smoking status	Yes	0.272***	1.312	(1.086, 1.585)	0.256*	1.292	(0.992, 1.681)	0.206	1.229	(0.956, 1.580)	0.735***	2.085	(1.206, 3.606)
Alcohol status	Yes	0.216**	1.241	(1.052, 1.463)	0.154	1.166	(0.934, 1.457)	0.354***	1.425	(1.137, 1.785)	−0.128	0.880	(0.512, 1.512)
Sleep duration	6–8 h	−0.790***	0.454	(0.381, 0.542)	−0.901***	0.406	(0.330, 0.499)	−0.596***	0.551	(0.424, 0.717)	−0.934***	0.393	(0.228, 0.676)
	≥8 h	−0.828***	0.437	(0.327, 0.583)	−1.374***	0.253	(0.167, 0.384)	−0.430**	0.651	(0.433, 0.979)	0.000	1.000	(0.491, 2.039)
Marital status	Married	−0.083	0.920	(0.697, 1.214)	−0.188	0.829	(0.591, 1.163)	−0.003	0.997	(0.652, 1.525)	0.171	1.186	(0.467, 3.020)
Living alone	Yes	−0.028	0.972	(0.719, 1.314)	−0.113	0.893	(0.614, 1.299)	−0.076	0.927	(0.587, 1.463)	0.678	1.970	(0.770, 5.039)
Monthly income	3,000–7,000 yuan	−0.122*	0.885	(0.771, 1.015)	−0.065	0.937	(0.784, 1.119)	−0.134	0.875	(0.715, 1.069)	−0.454**	0.635	(0.412, 0.980)
	≥7,000 yuan	0.329***	1.389	(1.146, 1.683)	0.332***	1.394	(1.096, 1.772)	0.458***	1.581	(1.181, 2.115)	−0.273	0.761	(0.368, 1.574)
Education level	Vocational/high school	−0.135	0.874	(0.741, 1.030)	−0.121	0.886	(0.712, 1.102)	−0.243**	0.784	(0.629, 0.978)	0.452	1.571	(0.927, 2.664)
	College diploma	−0.139	0.870	(0.720, 1.050)	−0.123	0.884	(0.691, 1.132)	−0.270*	0.763	(0.582, 1.002)	0.436	1.547	(0.839, 2.848)
	Bachelor’s degree or above	−0.149	0.862	(0.704, 1.056)	−0.030	0.970	(0.749, 1.259)	−0.370**	0.691	(0.511, 0.933)	−0.273	0.761	(0.367, 1.581)
Occupation	Manual laborer	0.179**	1.196	(1.022, 1.400)	0.252**	1.287	(1.044, 1.585)	0.083	1.087	(0.877, 1.346)	0.084	1.088	(0.668, 1.770)
	Non-manual worker	0.039	1.040	(0.902, 1.199)	0.191**	1.210	(1.012, 1.448)	−0.180	0.835	(0.669, 1.042)	−0.247	0.781	(0.483, 1.265)

*p<0.05; **p<0.01; ***p<0.001.

## 4 Discussion

### 4.1 Chronic disease patients exhibit heterogeneity in disease patterns

The latent class analysis results of this study indicate that the comorbidity patterns among patients with chronic diseases in Yantai City can be categorized into four latent classes: the low comorbidity group (82.39%), the musculoskeletal-chronic disease mixed group (9.48%), the metabolic syndrome-dominant group (7.01%), and the high comorbidity-complex group (1.12%).

In terms of distribution, the low comorbidity group (C1) is absolutely dominant, indicating that most patients with comorbidities are in a relatively simple disease combination stage, primarily suffering from 1 to 2 chronic diseases, such as isolated hypertension or diabetes combined with arthritis. The management focus for these patients lies in standardized control of underlying diseases and primary prevention of complications (16), with their health status still within a relatively favorable range amenable to intervention. The musculoskeletal-chronic disease mixed group (C2) is characterized by the coexistence of musculoskeletal disorders (e.g., osteoarthritis, osteoporosis) with other common chronic conditions. These patients often face multiple challenges including chronic pain, functional limitations, and systemic disease symptoms, significantly impacting their independence and mental health. They have an urgent need for rehabilitation services and social support (17). The Metabolic Syndrome-Dominant Group (C3) prominently exhibits clustering of core metabolic disorders such as hypertension, hyperglycemia, and dyslipidemia. This group represents an extremely high-risk cohort for future cardiovascular events (e.g., myocardial infarction, stroke) and kidney disease. Comprehensive, intensive lifestyle interventions and coordinated pharmacotherapy are urgently needed to break the vicious cycle of metabolic dysfunction (18). Although the high comorbidity-complex group (C4) represents the smallest proportion, its disease combinations are highly heterogeneous and complex, typically involving coexisting chronic diseases affecting three or more systems, presenting the most severe health challenges. Patients in this group often experience polypharmacy, frequent hospitalizations, functional dependency, and extremely high mortality risks. They constitute the core cohort that consumes the most healthcare resources and most urgently requires multidisciplinary collaboration, integrated care, and palliative support (19).

### 4.2 Gender and age significantly influence chronic disease comorbidity grouping among Yantai residents

The study findings reveal marked heterogeneity in the influence of gender across different disease comorbidity groups. In the musculoskeletal-chronic disease mixed group C2, males were a significant protective factor (OR = 0.664, 95% CI 0.552–0.799), potentially due to higher androgen levels in males offering protective effects on cartilage and bone (20, 21); In the metabolic syndrome-dominant group C3, males transitioned to a risk factor (OR = 1.745, 95% CI 1.440–2.116). This may stem from males’ tendency to accumulate fat in the abdominal region, forming abdominal obesity, also known as visceral fat (22). This type of fat is associated with higher metabolic risk because it is more active, releasing fatty acids into the bloodstream, leading to insulin resistance and dyslipidemia (23, 24).

This study indicates that age is a common risk factor across all four categories. With advancing age, the body undergoes irreversible organ function decline, reduced cellular repair capacity, and immune aging, providing a shared physiological foundation for the development of multiple chronic diseases (25, 26). Consistent with findings from numerous comparable studies—such as surveys of middle-aged and older adult(s) populations in Shanghai, Guangzhou, Xinjiang, and other regions—age has been identified as the strongest predictor of comorbidity (27–29). As an economically developed coastal city with a high life expectancy, Yantai’s seventh national census revealed that its population aged 65 and above accounted for 18.12%. This indicates that Yantai faces a more severe challenge of longevity without health—a large middle-aged and older adult(s) population living with chronic conditions for extended periods, bearing a heavy burden of co-morbidities.

Therefore, Yantai’s policy development must exhibit high age-specific targeting and stratification awareness for comorbidities. First, leveraging its robust primary healthcare network, the city should mandate screening for comorbidities and risk assessment as core components of basic public health services. Dynamic health records should be established for older adult(s) residents across different comorbidity groups, transitioning from generic geriatric checkups to precision interventions based on comorbidity risk. Second, addressing the gender heterogeneity identified in the study, differentiated health promotion campaigns should be implemented in communities. Women should be encouraged to engage in weight-bearing exercises to maintain bone health, while men should receive targeted interventions focusing on abdominal obesity management, smoking cessation, alcohol reduction, and regular monitoring of metabolic indicators.

### 4.3 Health and unhealthy behaviors significantly influence chronic disease comorbidity grouping among Yantai residents

Appropriate exercise and adequate sleep serve as common protective factors across all four categories. Consistent with extensive evidence from prospective cohort studies worldwide, regular exercise improves cardiovascular function, controls weight, enhances insulin sensitivity, and alleviates anxiety and depression. These effects collectively reduce the risk of multiple chronic diseases including hypertension, diabetes, and cardiovascular/cerebrovascular diseases through multiple pathways (30–32). Adequate sleep is crucial for endocrine regulation, immune system stability, and cognitive function maintenance (33). Chronic sleep deprivation is an independent risk factor for metabolic syndrome and mental health issues (34).

Excessive consumption of pickled foods is a common risk factor across all four categories. As a major maritime city, Yantai’s culinary culture traditionally features pickled seafood. However, such foods are rich in nitrosamine compounds and high sodium concentrations. Nitrosamines are potent carcinogens clearly associated with gastrointestinal tumor risks (35); high sodium intake is a primary cause of hypertension, stroke, gastric cancer, and kidney damage (36).

Alcohol consumption is a risk factor for the dominant metabolic syndrome group C3. Excessive alcohol intake directly damages hepatocytes, leading to fatty liver disease, alcoholic hepatitis, cirrhosis, and even liver cancer (37). Additionally, alcohol itself is a high-calorie empty-calorie substance that readily contributes to obesity and disrupts blood pressure and lipid metabolism (38). In the C3 group with existing metabolic disorders, alcohol’s negative pharmacological effects on blood glucose, lipids, and blood pressure are dramatically amplified, highlighting its inherent risks (24).

Therefore, it is recommended that Yantai’s public policy formulation adopt a strategy combining universal advocacy with targeted interventions. At the universal level, comprehensive health education should be vigorously promoted through community and media channels, emphasizing the importance of regular exercise and adequate sleep. Concurrently, local dietary culture should be actively guided toward transformation by developing and promoting low-salt curing techniques and establishing “low-salt specialty food” labels, thereby reducing health risks while preserving traditional flavors. At the targeted level, high-risk populations with comorbidities should be prioritized for intervention. For residents already exhibiting metabolic abnormalities, health education must emphasize the dangers of alcohol consumption and provide strict recommendations for limiting or abstaining from alcohol.

### 4.4 Income level, educational attainment, and occupational type exert group-specific effects on the clustering of chronic diseases among Yantai residents

Higher average monthly income is a risk factor for the musculoskeletal-chronic disease mixed group C2 and the metabolic syndrome-dominant group C3. While higher income is typically associated with better healthcare and living conditions, it may also be accompanied by unhealthy dietary habits (such as consumption of high-fat, high-sugar foods) and sedentary lifestyles, thereby increasing the risk of metabolic syndrome (39). Osteoarthritis (OA), a common joint disorder causing chronic pain and disability, may be more prevalent among high-income individuals due to sedentary occupations that limit physical activity, potentially increasing musculoskeletal disease risk (40).

Compared to the unemployed/other category, manual laborers and non-manual workers represent risk factors for the musculoskeletal-chronic disease mixed group. Manual workers typically face higher physical stress and repetitive tasks, which may elevate musculoskeletal disease risk (41, 42). In contrast, non-manual workers may encounter distinct health hazards from poor posture and repetitive motions (43). Unfavorable work environments—such as ill-fitting furniture and poorly ventilated offices—also negatively impact non-manual workers’ health (44).

Therefore, Yantai’s public health policies should transcend traditional socioeconomic perspectives and implement more targeted, precise interventions. First, for high-income groups, prioritize risk communication and behavioral guidance regarding lifestyle choices, advocating balanced diets, regular exercise, and stress management to counteract the negative effects of high-income-associated high stress, high-calorie intake, and sedentary lifestyles. Second, implement tiered occupational health management: for manual workers, strengthen enforcement of the Occupational Disease Prevention and Control Law, promote workplace exercises, provide ergonomic equipment, and enhance primary prevention of musculoskeletal disorders; for non-manual workers, integrate metabolic syndrome screening and intervention into corporate health cultures, advocate standing desks, and organize regular physical activities. Ultimately, policies should foster societal consensus that health is the greatest wealth, enabling all income and occupational groups to prioritize health management and thereby reduce Yantai’s overall burden of co-morbidities.

### 4.5 Research limitations

This study has several limitations that should be carefully considered when interpreting the findings. First, the study design is cross-sectional, with data sourced from a single-point field survey conducted in Yantai City in 2024. This prevents us from establishing causal relationships between variables, revealing only associations. Additionally, chronic disease prevalence information relies primarily on self-reported data from respondents. While a common method, this approach may introduce bias compared to clinical diagnostic records, potentially affecting the accuracy of results. Second, the study employed latent class analysis to explore comorbidity patterns. While this method effectively identifies hidden subgroup structures within data, its results are model-dependent. Different datasets or varying model parameter settings may yield divergent category solutions, potentially compromising the stability and reproducibility of findings. Third, the convenience sampling method employed resulted in a sample age structure heavily concentrated among middle-aged and young adults, failing to adequately cover the older adult(s) population. This limited representativeness of the sample to the general population may have significantly underestimated the overall prevalence and severity of comorbidities, restricting the generalizability of conclusions to the entire population—particularly high-risk older adult(s) cohorts. Finally, the identified “high-comorbidity-complex group” constituted an extremely low proportion (1.12%) of the sample. This small proportion resulted in weak statistical power for this subgroup, with wide confidence intervals for risk factor estimates (e.g., OR values), casting doubt on their stability. It remains unclear whether this group represents a clinically distinct entity or merely statistical noise from model fitting. Its clinical interpretability requires further validation in larger cohorts, especially those with more older adult(s) individuals.

## Data Availability

The raw data supporting the conclusions of this article will be made available by the authors, without undue reservation.
